# An Incidental Finding of Retrocaval Ureter Causing Hydroureteronephrosis

**DOI:** 10.7759/cureus.28973

**Published:** 2022-09-09

**Authors:** David N Ray, Natalie F Smith, Jorge Quiros, Chelsey Thachettu, Wilfred McKenzie

**Affiliations:** 1 Medicine, Meharry Medical College, Nashville, USA; 2 Medicine, Nova Southeastern University College Of Osteopathic Medicine, Fort Lauderdale, USA; 3 Internal Medicine, Broward Health, Fort Lauderdale, USA

**Keywords:** computed tomography (ct) imaging, hydronephrosis, acute gastrointestinal bleed, inferior vena cava anomaly, retrocaval ureter

## Abstract

Retrocaval ureter is a rare, congenital anomaly of the inferior vena cava. Due to an abnormal process in embryogenesis, the ureter descends posterior to the inferior vena cava. Although the anomaly can be found in pediatric patients, it most commonly manifests symptoms between the third and fourth decade of life that are typically due to ureteric obstruction, such as hydronephrosis. Retrocaval ureter can be asymptomatic and may be the reason for unreported cases and low incidence in the world. In this case, we reviewed the record of a patient with the diagnosis of retrocaval ureter incidentally found during admission for a lower gastrointestinal bleed.

## Introduction

Circumcaval ureter, more commonly known as retrocaval ureter, is a rare congenital anomaly with fewer than 200 cases being reported since first described in 1893 [1]. In this anomaly, the proximal ureter travels posterior to the inferior vena cava (IVC) and eventually inserts at the renal pelvis. The ureter becomes trapped posteriorly due to an abnormal formation of the infrarenal IVC from the ventrally located subcardinal vein. The infrarenal IVC normally originates dorsally from the supracardinal vein between the fourth and eighth week of intrauterine development [1]. Normally, the distal ureter travels superficial to the common iliac vessels prior to insertion at the bladder [2]. Although the anomaly is present at birth, patients are typically asymptomatic until the third or fourth decades of life due to IVC compression of the proximal ureter leading to hydronephrosis or hydroureteronephrosis [3].

In this case report, we present a patient who arrived at the emergency department (ED) for dark stools for two days, and a retrocaval ureter was seen on computed tomography (CT) of the abdomen and pelvis as an incidental finding. Although the patient had mild hydronephrosis secondary to his retrocaval ureter, he remained asymptomatic.

## Case presentation

History of presentation

A 55-year-old African American male with a history of hypertension and chronic alcohol use disorder complained of painless, burgundy-colored bowel movements for three days associated with chronic, intermittent lower back pain. He denied difficulty initiating urination, hesitancy, or urgency despite his enlarged prostate. He denied any medication use besides occasional aspirin. He had an extensive alcohol intake history. On physical examination, the patient was hypertensive (171/103), afebrile, had a regular heart rate, and was in no acute distress. His abdomen was soft, nontender, and nondistended with normal bowel sounds.

Investigations

The patient’s complete blood count (CBC), chemistry panel (CMP), and liver function tests were relatively unremarkable, but he was found to have a positive fecal occult blood test and microscopic hematuria. His ethanol level was less than 10 mg/dL. His electrocardiogram on arrival revealed normal sinus rhythm with a heart rate of 73 beats/minute. Management in the ED included 1 L bolus of normal saline gastrointestinal prophylaxis and a CT of the abdomen and pelvis with intravenous (IV) contrast.

**Figure 1:**
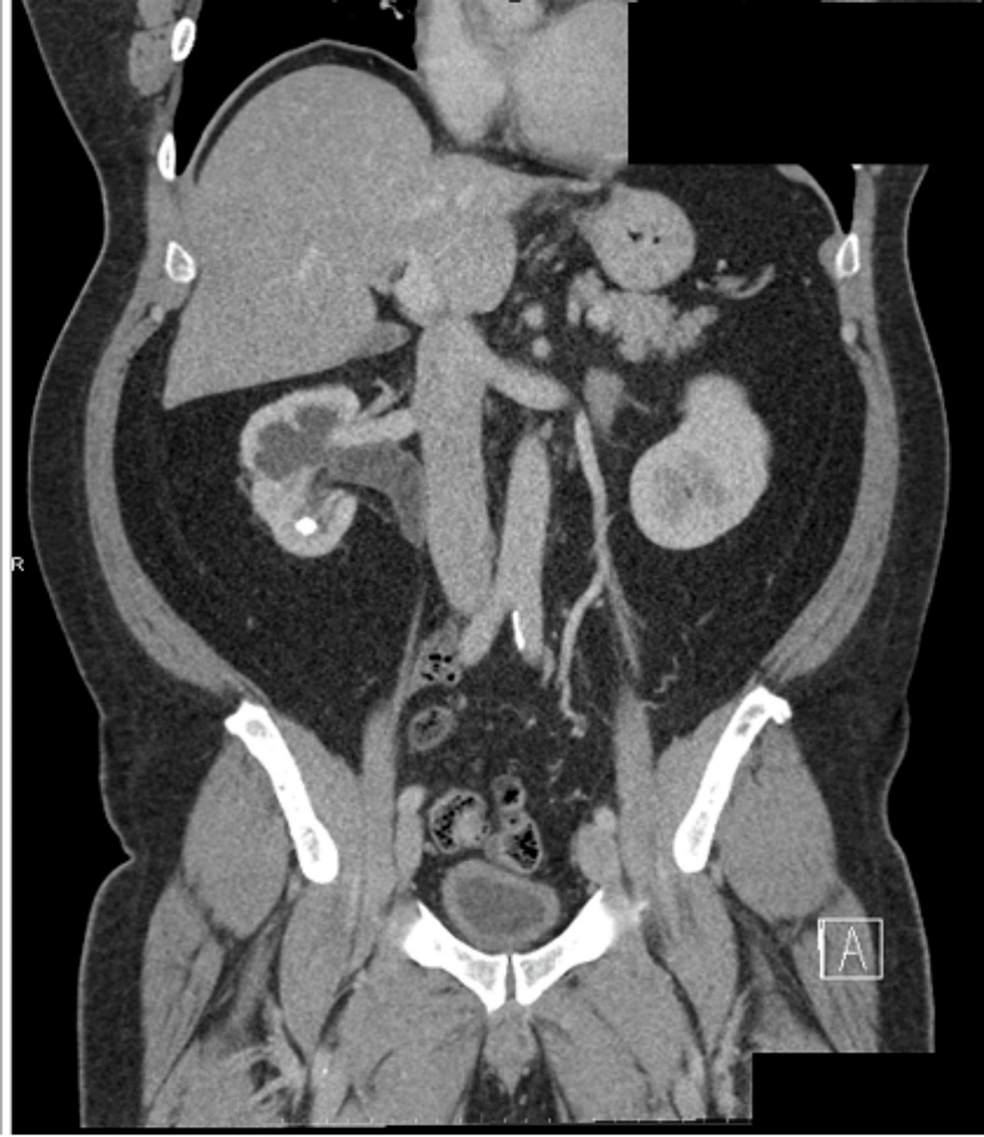
Coronal view of computed tomography of the abdomen/pelvis with intravenous contrast. Retrocaval ureter with incidental nephrolithiasis.

**Figure 2:**
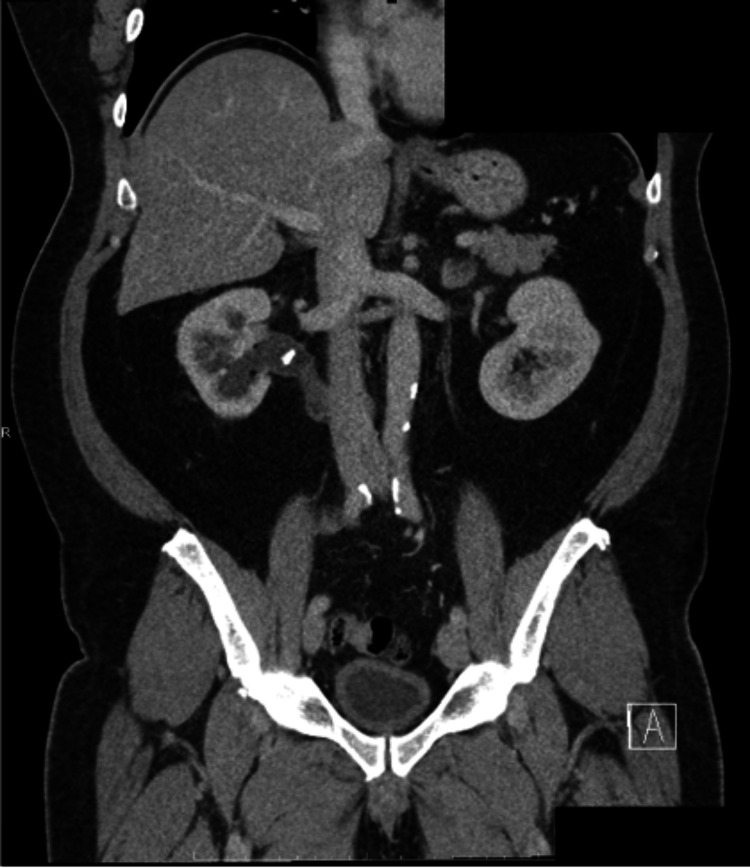
Coronal view of computed tomography of the abdomen/pelvis with intravenous contrast. Retrocaval ureter with incidental ureterolithiasis.

CT of the abdomen and pelvis with IV contrast revealed moderate proximal right hydroureteronephrosis likely as a result of compression of the proximal right ureter from the IVC, more commonly known as a retrocaval ureter. There was also a nonobstructive 8 mm calculus in the inferior pole of the right kidney (Figure 1) and a nonobstructive 7 mm calculi in the proximal right ureter (Figure 2). There was also colonic diverticulosis without diverticulitis, which was the most likely source of his gastrointestinal bleeding. Lastly, the CT revealed a thick-walled urinary bladder and an enlarged prostate gland, prompting the order for a prostate-specific antigen level, which was 0.6 ng/mL.

Management

The patient was primarily admitted to the hospital for managing his lower gastrointestinal bleed, but further intervention was required due to the incidental findings of a retrocaval ureter anomaly causing hydroureteronephrosis. The repeated CBC and CMP over the next few days remained unremarkable. A colonoscopy revealed internal hemorrhoids and severe diverticulosis with active bleeding of the diverticulum, which was controlled by applying two hemostatic clips. Per urology, definitive reconstructive surgery and stone removal can be performed simultaneously by a reconstructive urologist in the outpatient setting. He was discharged on tamsulosin to manage his new diagnosis of retrocaval ureter.

## Discussion

There are two classifications of retrocaval ureters, namely, type one and type two. In type one, the ureter travels posterior to the IVC at the level of the third lumbar vertebra and presents as an S- or a fish-hook-shaped deformity presenting with subsequent moderate-to-severe hydronephrosis. Type two is less common, and it is depicted by the proximal ureter traveling posterior to the IVC at the same level of the renal pelvis forming a smooth, sickle-shaped curve [3-5]. Hydroureteronephrosis is defined as an obstruction to free-flowing urine complicated by dilation of the ureter, renal pelvis, and renal calyces. In severe cases, it can lead to atrophy of the renal cortex [6].

The clinical presentation of a symptomatic patient is most commonly described as obstructive uropathy symptoms, such as right flank pain, recurrent urinary tract infections, nephrolithiasis, and hematuria [7]. On a review of previous case reports of symptomatic patients with retrocaval ureter in Ghana, they both had chronic flank pain, a normal laboratory evaluation, and right-sided hydronephrosis on CT [1]. Neither case report described the severity of the hydronephrosis, but they both required excision of the retrocaval segment with end-to-end ureteral anastomosis [1]. The patient in this case had bilateral nonobstructive urinary calculi without flank pain. He had chronic, intermittent lower back pain, although it was attributed to his manual labor job. Prostatic carcinoma with metastasis to the spine was considered due to his back pain and asymptomatic enlarged prostate, but he had a prostate-specific antigen level of 0.6 ng/mL and a normal alkaline phosphatase level of 54 U/L.

There are multiple modalities in which imaging can be used to diagnose a retrocaval ureter. The method of choice for diagnosing retrocaval ureter is CT urography because it is the most efficacious and least invasive [8]. It provides visualization of the anatomical relationships between the ureter and IVC. However, some literature suggests that spiral CT scan is the superior option because it can outline both the ureter and IVC [1]. Magnetic resonance imaging is equivalent to CT imaging and does not provide radiation risk or renal damage due to iodine toxicity [9]. A nuclear renal scan can be helpful in evaluating the severity of obstruction. Ultrasound can provide visualization of pyelo-calyceal dilatation of the kidney [8,9].

Asymptomatic patients with mild-to-moderate hydronephrosis can be managed without surgical intervention; however, symptomatic patients with severe hydronephrosis and with worsening kidney function require surgical treatment [10]. There are numerous ways for grading hydronephrosis. The radiology grading system assesses parenchymal loss as a measure of severity, whereas the Society of Fetal Urology (SFU) assesses renal pelvic dilation as a measure of severity. Although SFU is most commonly used, it is mostly used to assess neonatal and infant pelvicalyectasis [11]. The Onen grading system measures the dilation of the pelvicalyceal system and the quality of renal parenchyma, which takes into account renal damage. This grading system has parameters that have been proven to be reproducible and standardized [11].

Retroperitoneal laparoscopic ureteroplasty in symptomatic patients with retrocaval ureter is recommended as the first choice in corrective surgery due to its safety and effectiveness, faster recovery, decrease in blood loss, shortened hospital visits, and decreased postoperative pain [12]. A case series involving two patients with retrocaval ureter receiving laparoscopic surgery reported the operative time was 210 and 180 minutes without significant blood loss and a postoperative hospital stay of fewer than two days [8]. The repair typically involves resection of the redundant retrocaval ureteral segment, anteposition, and ureteroureteral or ureteropelvic anastomosis [13].

## Conclusions

This case highlights a unique presentation of clinically asymptomatic, moderate hydronephrosis due to retrocaval ureter incidentally found on CT imaging. Preoperative diagnosis is best established through a CT scan. The standard of care to correct this anomaly is minimally invasive laparoscopic surgery; however, asymptomatic patients should be managed conservatively. Retrocaval ureter is a rare anomaly, but the number of cases reported is slightly increasing. The cause for this is most likely multifactorial, which could include improvement in radiographic imaging, increased access to healthcare, or simply an increased number of reports by physicians. Nevertheless, it is important to consider retrocaval ureter in a patient with asymptomatic hydronephrosis, despite a low index of suspicion for this disease.
